# Flora-On: Occurrence data of the vascular flora of mainland Portugal

**DOI:** 10.3897/phytokeys.69.9432

**Published:** 2016-09-09

**Authors:** Ana Júlia Pereira, Ana Francisco, Miguel Porto

**Affiliations:** 1 Sociedade Portuguesa de Botânica (SPBotânica), Travessa do Jardim n° 3, A-dos-Potes, 2615-018 Alverca, Portugal

**Keywords:** Portugal, vascular plants, occurrence, observation

## Abstract

The Flora-On dataset currently includes 253,310 occurrence records for the class Embryopsidae (vascular plants), comprising data collated via the platform http://flora-on.pt/ relating to observation records of vascular plants across mainland Portugal. Observations are uploaded directly to the database primarily by experienced botanists and naturalists, typically on a weekly basis, and consist of geo-referenced data points for species (or infraspecific taxa) along with their date of observation and phenological state.

The Flora-On project aims to compile and make publicly accessible chorological, ecological, morphological and photographic information for the entire vascular flora of Portugal. The project’s website offers powerful query and visualization capabilities, of which we highlight the probabilistic bioclimatic and phenological queries which operate based on the empirical density distributions of species in those variables.

Flora-On was created and continues to be maintained by volunteers who are Associate members of Sociedade Portuguesa de Botânica (Botanical Society of Portugal). Given its focus on research-grade and current data, the Flora-On project represents a significant contribution to the knowledge of the present distribution and status of the Portuguese flora.

## Project details

### Project title

Flora-On, Interactive Flora of Portugal

### Personnel

Miguel Porto (Programmer)

### Funding

The project does not have direct funding from any source, the platform being entirely built and maintained by volunteers. Maintenance costs of the web server are covered by the Associate membership fees of Sociedade Portuguesa de Botânica (Botanical Society of Portugal). However, externally funded projects have contributed through the provision of data.

### Study area description

**Figure 1.**
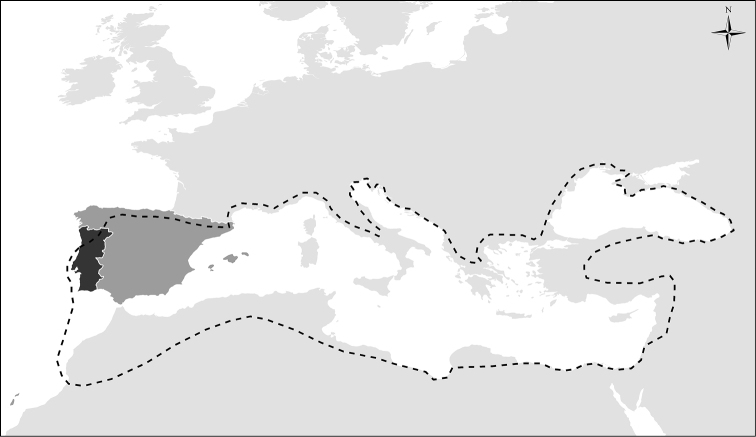
Location of mainland Portugal (black) which, together with Spain (dark grey), forms the Iberian Peninsula. Dashed line represents the boundary of the Mediterranean macrobioclimate, which contacts with the temperate macrobioclimate to the north.

Portugal is located at the south westernmost extent of Europe (Figure 1) and is bound by the Atlantic Ocean to the west and south and by Spain to the north and east. Being approximately rectangular in shape, Portugal extends circa 220 km from east to west and 550 km from north to south. It lies within the Mediterranean biogeographic region, with the vast majority of its land falling within the Mediterranean macrobioclimate but extends in the north into the temperate macrobioclimate. The area of mainland Portugal is approximately 89,015 km^2^ and, together with mainland Spain (492,127 km^2^), forms a geographically well-defined territory known as the Iberian Peninsula.

The orography of Portugal is heterogeneous, particularly from north to south, with the Mountains and Plains of the Iberian northwest and of the Iberian Central System dominating the northern parts of its territory (Pereira et al. 2014). This region is characterised by rugged landscapes dominated by granitic and metasedimentary geological formations which extend almost as far south as river Tejo (the largest river in Portugal which divides the country in half). Serra da Estrela mountain, its highest peak, rises 1,991 m above sea level. The Plains of the southwest Iberian Peninsula occupy almost all the central and southern interior territory (Pereira et al. 2014), this region being characterised by a very smooth landscape with some scattered high relief formations, such as Serra de São Mamede (1027 m), Serra de Monchique (902 m), and Serra do Caldeirão (589 m). The western and southern coastal regions are otherwise occupied by the Mesozoic and Cenozoic Basins (Pereira et al. 2014) and are characterised by the dominance of maritime and alluvial sedimentary formations and calcareous reliefs, some of which are very prominent, including Serra de Montejunto (666 m), Maciço Calcário Estremenho (610 m), Serra da Arrábida (499 m), and Barrocal Algarvio (479 m).

Across mainland Portugal the vegetation is mainly Mediterranean in terms of both its structure and floristic composition. Semi-deciduous and perennial oak woodlands, “montado”, shrublands, grasslands and silvo-agricultural systems occupy most of this area. Mainland Portugal supports approximately 2,900 native vascular plant taxa (Sequeira et al. 2010), 137 of which are considered endemic.

### Design description

The Flora-On project aims to compile and make publicly accessible chorological, ecological, morphological and photographic information of the entire vascular flora of Portugal. Occurrence data is regularly uploaded to the website by active collaborators, typically on a weekly basis, and consists of geo-referenced data points of species (or infraspecific taxa) along with their date of observation and phenological state. Additionally, other research projects contributed data to the project from their exhaustive sampling campaigns. An open-source version of the platform is currently under development and can be found at https://github.com/miguel-porto/flora-on-server/

**Figure 2.**
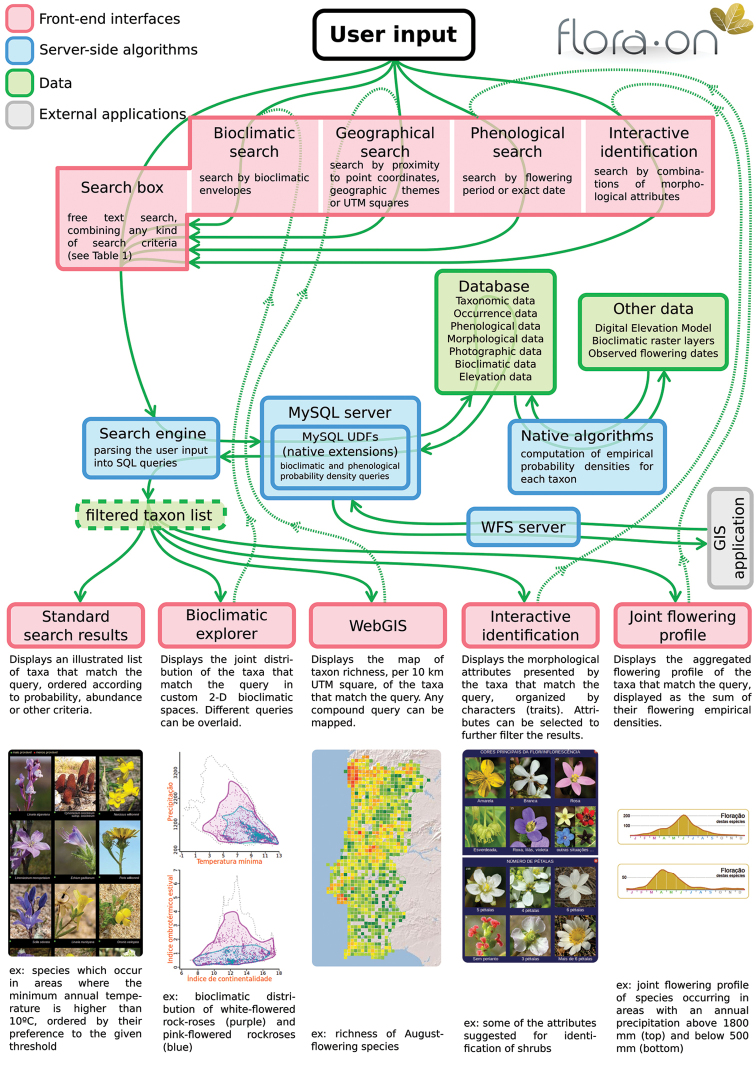
Internal structure, data flow and front-end interfaces of Flora-On. Pink boxes represent the front-end interfaces that interact with the user (input and/or output). Green boxes represent the data, either permanent or temporary (dashed box). Blue boxes represent the internal server-side algorithms that parse the user queries, process and summarise the raw data, and deliver the results to the front-end interfaces.

The strength of the Flora-On platform lies in its ability to execute a diversity of query types (Table 1). In addition to the usual deterministic queries in relation to taxonomic, morphological and geographical information, Flora-On enables users to conduct quantitative probabilistic species queries in relation to bioclimatic distribution, altitudinal distribution and flowering dates. With such queries species can be filtered and ranked by the degree of matching criteria defined by the user for one or more quantitative variables (including flowering date). This innovative feature is based upon empirical density distributions of species that are computed internally for each variable (Figure 2), and for the precise observed flowering dates, using a kernel smoother. The density distributions are stored in the database as binary objects to allow fast querying with MySQL native extensions.

**Table 1.** Example of query types which can be entered by users into the Flora-On search box. Any comma-separated combination of queries can be entered into the search box. Currently, Flora-On only accepts queries in Portuguese. The table provides the English translation in parentheses.

Type of query	Example queries (meaning)	Returns
Morphological	arbusto espinhoso, flores amarelas (spiny shrub, yellow flowers)	All taxa that present the specified attribute combination
Bioclimatic range	continentalidade>14 (continentality index greater than 14)	All taxa whose cumulative density distribution in each variable, within the specified ranges, is greater than a given threshold
	tempminima: 1.4-3.7, precipitação: 1300–1900 (minimum temperature between 1.4 and 3.7°C and annual precipitation between 1,300 and 1,900 mm)	
Bioclimatic similarity	tempmaxima~Cistus albidus (maximum temperature profile similar to that of *Cistus albidus*)	All taxa whose density distribution of the observations in the specified variable is similar to that of the specified species, given a threshold of similarity
Geographical variable ranges	altitude<100, costa>100000 (altitude lower than 100 m and distance to coast greater than 100 km)	All taxa whose cumulative density distribution in each variable, within the specified ranges, is greater than a given threshold
Geographical themes	Algarve (existing within Algarve region)	All taxa with at least one observation within the boundaries of the specified region, natural park, county, UTM square, or other geographical theme
	NC06 (existing in NC06 UTM square)	
Geographical proximity	perto: 38.4278 -9.1949 (close to 38.4278° N -9.1949° W)	Taxa having at least one observation within a given radius from the specified coordinate
Geographical similarity	distribuição~Staehelina dubia (distribution similar to that of *Staehelina dubia*)	Taxa with a distribution similar to that of *Staehelina dubia*, computed as the intersection of their density distributions, given a threshold of similarity
Phenological range	20 julho a 9 agosto (20 July to 9 August)	All taxa whose cumulative density distribution of flowering dates within the specified range is greater than a given threshold, i.e., taxa whose flowering period is concentrated within the specified range
Phenological similarity	floração~Scilla autumnalis (flowering profile similar to that of *Scilla autumnalis*)	All taxa whose density distribution of flowering dates is similar to that of *Scilla autumnalis*, given a threshold of similarity
Phenological precise date	7 fevereiro (7 February)	All taxa which may be found in flower at the given date (regardless of the flowering distribution throughout the year)
Area of occupancy	quadriculas<3 (less than 3 UTM squares)	All taxa that occur only in less than three 10×10 km UTM squares

Flora-On is designed in such a way that the results of any type of query, irrespective of its complexity, can be visualized across different facets, evidencing aggregated bioclimatic, geographical, phenological or morphological features of the species that match the query. All queries can be expressed through plain text, but to simplify the querying process for general users, four front-end graphical query interfaces are provided to aid query building (Figure 2, top row). The query algorithm, after passing and processing the input query (Figure 2, middle row), then delivers the results to the output modules of the application, which summarise and display the query results according to the different facets (Figure 2, bottom row):

- The standard search displays the species photographs ordered by different criteria;
- The bioclimatic explorer displays jointly the occurrences of the species that match the query in a bioclimatic/environmental space, with the possibility of overlaying multiple queries in the same plots, evidencing the ecological differences between species or groups of species (http://flora-on.pt/#b);
- The WebGIS displays (with the ability to download the output) the map of the number of species that match the query per Universal Transverse Mercator (UTM) square, e.g. richness of spiny species, richness of summer-flowering species, richness of species occurring in less than five UTM squares, etc. (http://flora-on.pt/#w);
- The multi-way interactive identification key allows users to identify species by iteratively narrowing down possible species, freely choosing its way through a set of characters. Displayed characters are adjusted for each iteration according to the list of possible species, and are highlighted according to their discriminant power, to enhance the efficiency of the identification process (http://flora-on.pt/#z);
- The joint flowering profile displays the aggregated flowering profile of all species matching the query, e.g. flowering profile of the species occurring in areas with an annual precipitation above 1,500 mm (currently accessible through the standard search results).

## Data published through

IPT: http://flora-on.pt:8080/ipt/resource.do?r=flora-on

GBIF: http://www.gbif.org/dataset/7fe3eb5c-42bd-49d7-a30b-82c353ef6575

Website: http://flora-on.pt/

## Taxonomic coverage

The Flora-On dataset includes 253,310 occurrence records of the class Embryopsidae. The top orders, comprising 75% of the records, include: Asterales (13.8%); Lamiales (11.3%); Poales (10.3%); Fabales (8.6%); Caryophyllales (6.5%); Asparagales (5.8%); Malvales (4.6%); Apiales (3.7%); Rosales (3.4%); Malpighiales (3.1%); Ericales (3%); and Fagales (2.7%).

**Figure 3.**
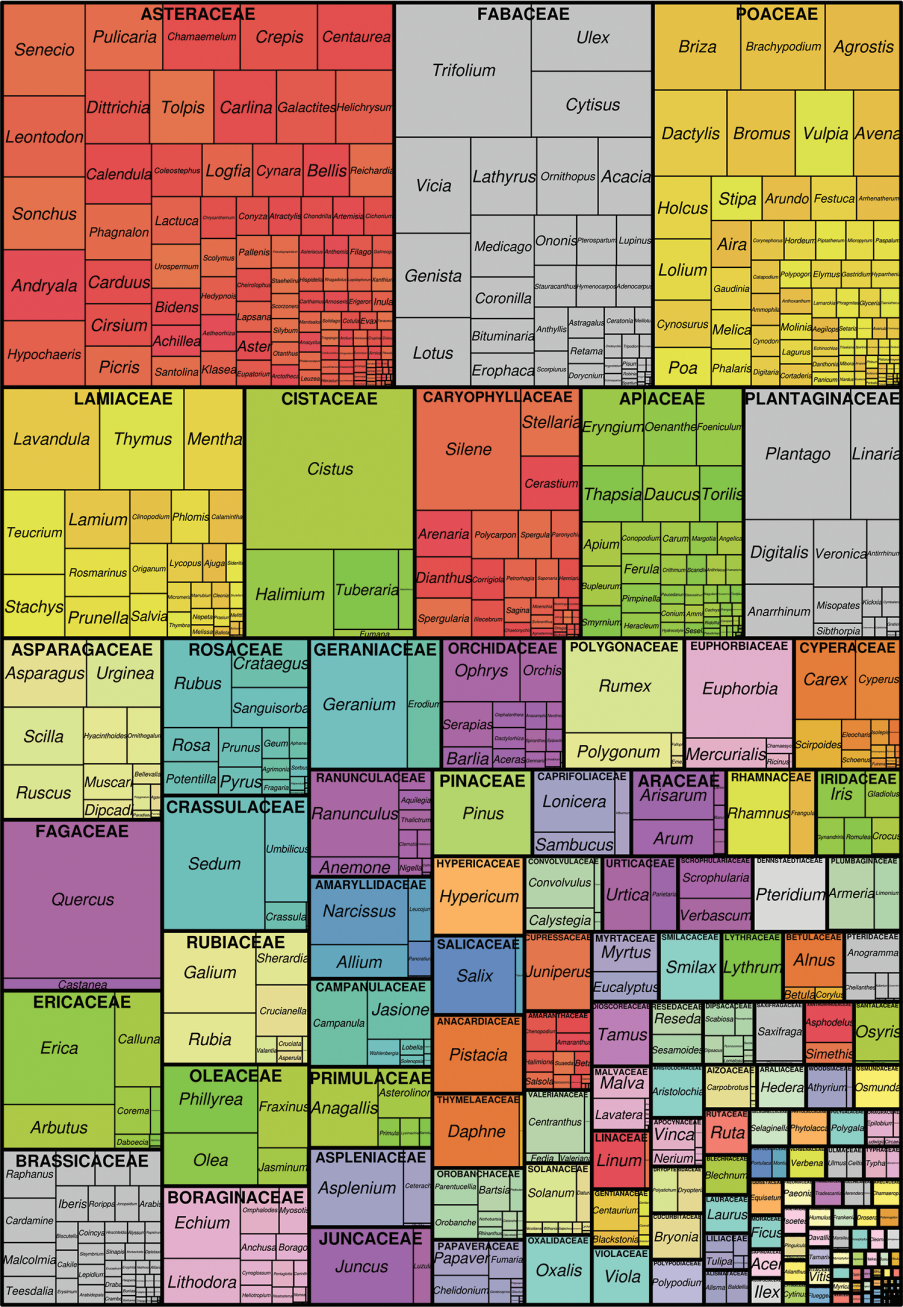
Tree map of the Flora-On dataset based on a two-level taxonomic classification. Families are delineated with thick lines and genera with thin lines. The size of the rectangles is proportional to the number of records held. The plot was built with the R package ‘treemap’ (Tennekes 2016).

In total, this dataset includes occurrence records for 150 plant families and 2073 taxa (Figure 3). Families with the greatest numbers of species in Portuguese mainland are also those families with the greatest number of occurrence records within this dataset, including: Asteraceae (32,638); Fabaceae (21,529); and Poaceae (20,656); although some genera are still under-represented. This is probably due to the nature of the dataset, given that the greatest part of the contributions results from non-exhaustive field observations which likely result in the under-representation of the more inconspicuous taxa, or taxa difficult to identify in the field.

## Taxonomic ranks

Kingdom: Plantae (Chlorobionta)

Phylum: Streptophyta

Class: Embryopsidae

Order: Alismatales, Apiales, Aquifoliales, Arecales, Asparagales, Asterales, Boraginales, Brassicales, Buxales, Caryophyllales, Celastrales, Ceratophyllales, Commelinales, Cornales, Cucurbitales, Cupressales, Cyatheales, Dioscoreales, Dipsacales, Ephedrales, Equisetales, Ericales, Fabales, Fagales, Gentianales, Geraniales, Hymenophyllales, Isoetales, Lamiales, Laurales, Liliales, Lycopodiales, Malpighiales, Malvales, Myrtales, Nymphaeales, Ophioglossales, Osmundales, Oxalidales, Pinales, Piperales, Poales, Polypodiales, Proteales, Ranunculales, Rosales, Salviniales, Santalales, Sapindales, Saxifragales, Selaginellales, Solanales, Vitales, Zygophyllales

Family: Acanthaceae, Aizoaceae, Alismataceae, Amaranthaceae, Amaryllidaceae, Anacardiaceae, Apiaceae, Apocynaceae, Aquifoliaceae, Araceae, Araliaceae, Arecaceae, Aristolochiaceae, Asparagaceae, Aspleniaceae, Asteraceae, Basellaceae, Betulaceae, Blechnaceae, Boraginaceae, Brassicaceae, Butomaceae, Buxaceae, Cactaceae, Campanulaceae, Cannabaceae, Caprifoliaceae, Caryophyllaceae, Celastraceae, Ceratophyllaceae, Cistaceae, Cleomaceae, Colchicaceae, Commelinaceae, Convolvulaceae, Cornaceae, Crassulaceae, Cucurbitaceae, Culcitaceae, Cupressaceae, Cynomoriaceae, Cyperaceae, Cytinaceae, Davalliaceae, Dennstaedtiaceae, Dioscoreaceae, Dipsacaceae, Droseraceae, Drosophyllaceae, Dryopteridaceae, Elaeagnaceae, Elatinaceae, Ephedraceae, Equisetaceae, Ericaceae, Euphorbiaceae, Fabaceae, Fagaceae, Frankeniaceae, Gentianaceae, Geraniaceae, Haloragaceae, Hydrangeaceae, Hydrocharitaceae, Hymenophyllaceae, Hypericaceae, Iridaceae, Isoetaceae, Juglandaceae, Juncaceae, Juncaginaceae, Lamiaceae, Lauraceae, Lentibulariaceae, Liliaceae, Linaceae, Linderniaceae, Lycopodiaceae, Lythraceae, Malvaceae, Marsileaceae, Melanthiaceae, Menyanthaceae, Molluginaceae, Moraceae, Myricaceae, Myrtaceae, Nartheciaceae, Nyctaginaceae, Nymphaeaceae, Oleaceae, Onagraceae, Ophioglossaceae, Orchidaceae, Orobanchaceae, Osmundaceae, Oxalidaceae, Paeoniaceae, Papaveraceae, Passifloraceae, Phyllanthaceae, Phytolaccaceae, Pinaceae, Pittosporaceae, Plantaginaceae, Platanaceae, Plumbaginaceae, Poaceae, Polygalaceae, Polygonaceae, Polypodiaceae, Pontederiaceae, Portulacaceae, Potamogetonaceae, Primulaceae, Proteaceae, Pteridaceae, Ranunculaceae, Resedaceae, Rhamnaceae, Rosaceae, Rubiaceae, Ruppiaceae, Rutaceae, Salicaceae, Salviniaceae, Santalaceae, Sapindaceae, Saxifragaceae, Scrophulariaceae, Selaginellaceae, Simaroubaceae, Smilacaceae, Solanaceae, Tamaricaceae, Taxaceae, Thelypteridaceae, Thymelaeaceae, Tropaeolaceae, Typhaceae, Ulmaceae, Urticaceae, Valerianaceae, Verbenaceae, Violaceae, Vitaceae, Woodsiaceae, Xanthorrhoeaceae, Zosteraceae, Zygophyllaceae

Common names: Vascular plants

## Spatial coverage

### General spatial coverage

**Figure 4.**
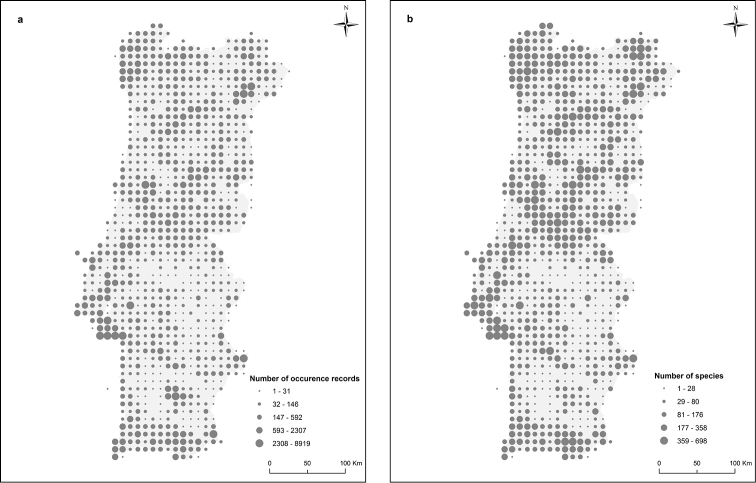
Number of occurrences (**a**) and species (**b**) recorded in Portugal mainland per 10×10 km square. The dataset used in this analysis includes a total of 253,310 records. Numbers were classified with geometrical intervals. The dots are placed at the centre of WGS84 UTM squares.

The Flora-On dataset covers almost the entire territory of mainland Portugal, although there remains a significant lack of information for some areas, particularly in the central and southern interior regions (Figure 4a). As expected for a dataset that is not complete, the number of species per UTM square (Figure 4b) correlates to the number of records, illustrating the gap in information for some regions. Indeed, whilst the project presently includes occurrence data spread across the whole country, the more intensively surveyed areas include the more coastal regions and some key areas towards the interior. Nonetheless, it is worth noting the high numbers of species of some 10x10 km UTM squares, up to 700 observed species in some cases, revealing high taxonomic diversity of some parts of the Portuguese mainland territory (Figure 4b).

**Figure 5.**
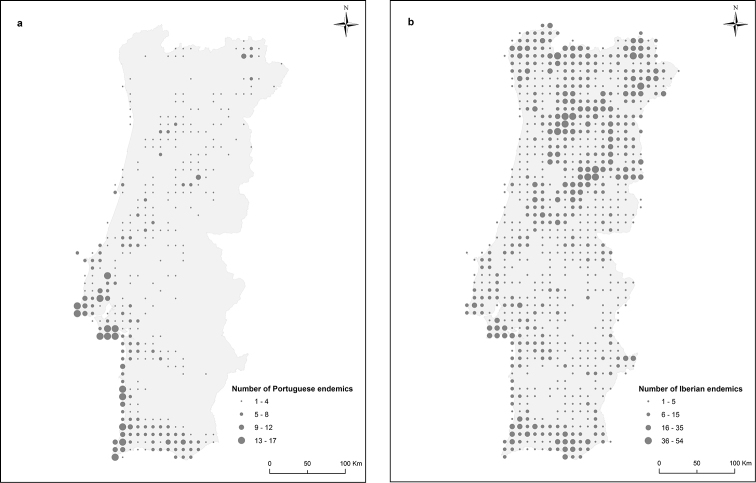
Number of Portuguese (**a**) and Iberian (**b**) endemic plants per 10×10 km UTM square. The dataset used in the analysis corresponds to a total of 253,310 records. Note that low values do not necessarily mean absence of endemic species, as many areas are under-sampled (Figure 4a). Class breaks are manual. The dots are placed at the centre of WGS84 UTM squares. N.B. Iberian endemics (**b**) do not include Portuguese endemics.

The number of Portuguese endemic species recorded per UTM square (Figure 5a) illustrates a well defined pattern, with the highest endemic species richness occurring across the central and southern coastal regions, including Lisbon and Setúbal, coast of Alentejo, and Algarve (from Sagres to Faro). These areas exhibit particularly isolated climatic and/or geological features, such as the wet coastal mountains of Sintra and Monchique, the inland sand plains of Setúbal, the Atlantic coastal cliffs, and the vast dry limestone regions of Setúbal and Algarve. Additionally, the data illustrates the importance of the mountain ranges in the interior north and the regions nearby the frontier in the northeast quadrant as areas of high Iberian endemic species richness (excluding Portuguese endemics) per UTM square (Figure 5b). Figure 5 further illustrates some coincidence between areas with high Portuguese endemic richness and high Iberian endemic richness.

### Coordinates

36°43'12"N and 42°10'12"N Latitude; 9°37'12"W and 6°9'36"W Longitude

### Temporal coverage

**Figure 6.**
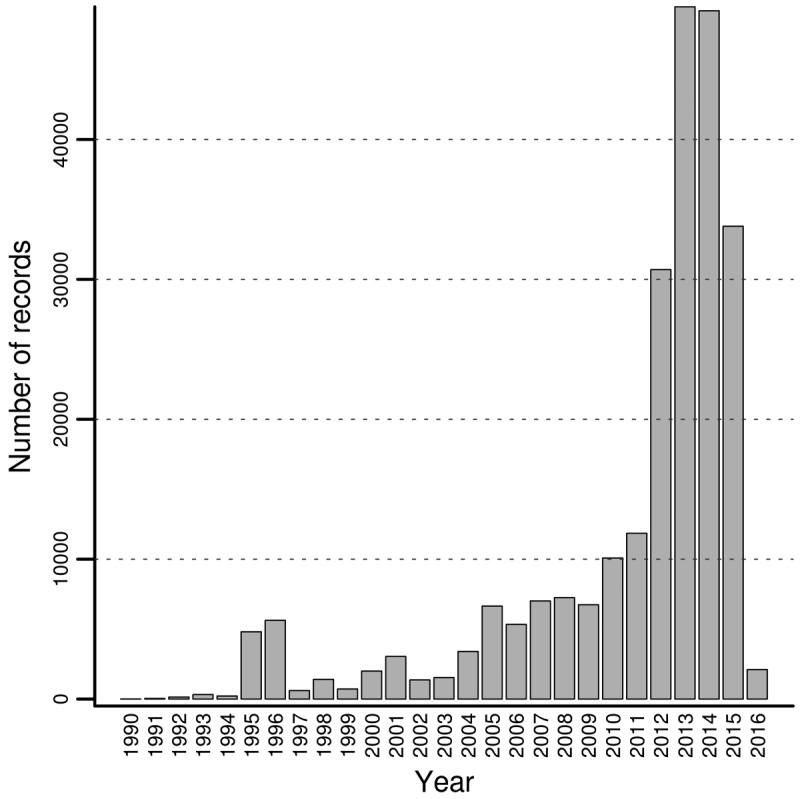
Barplot depicting the number of occurrence records observed per year. Occurrences observed before 1990 (127) were omitted for clarity. 7,681 records are not dated.

Although the bulk of the dataset corresponds to observations made between 1 January 1995 and 2 February 2016 (Figure 6), historic records prior to this period also exist.

### Collection name

Flora-On: Interactive Flora of Portugal

### Collection identifier

c9d06ad2-6369-47dd-9372-ade1a5055096

## Methods

### Method step description

For each occurrence, GPS coordinates are recorded by the collaborators wherever possible; otherwise approximate coordinates and their level of precision are recorded. Plants are identified at least to species level. Thereafter, collaborators upload the data to the Flora-On database via a webmapping interface or by uploading a record table. Coordinates are then generalised to the UTM 10×10 km grid and are made publicly available for download as tables and as a geographical layer through a WFS service: http://flora-on.pt/wfs. High resolution data can be provided upon request, subject to approval by Sociedade Portuguesa de Botânica and involved collaborators.

**Study extent description**: This dataset includes observations falling within mainland Portugal, most of which were made after the Flora-On platform was made available online (25 February 2012).

**Sampling Description**: A large proportion of the records corresponds to non-exhaustive observations of collaborators, although a significant amount of data results from fieldwork completed as part of other externally funded projects. When possible, plants are identified in the field at least to species level. Otherwise, plant material is collected and identification is confirmed in the lab by the collaborators. Phenological state is recorded if plants are flowering at the time of observation.

### Quality Control Description

Taxon nomenclature is fully controlled via use of a reference checklist, allowing neither spelling errors nor outdated synonyms. The reference checklist includes only currently accepted nomenclature which corresponds to an updated version of the “Checklist da Flora de Portugal (Continental, Açores e Madeira)” (http://ipt.gbif.pt/ipt/resource.do?r=alfa_checklist_florapt).

The responsibility of species identification rests with the collaborators, most of which have expertise in plant identification. Additionally, the Editorial Board of Flora-On is committed to ensure a high reliability of uploaded data, hence checking regularly for unlikely or doubtful occurrence records, and asking collaborators to provide pictures, descriptions or specimens whenever needed. The Editorial Board estimates at least 95% of the records to be correctly identified under the most up-to-date nomenclature.

## Datasets

The current Flora-On dataset published through GBIF includes occurrence and phenological data. Phenological data, for now, is limited to a ‘Yes’/’NA’ field in respect of flowering, and is linked to the date of the observation. In addition, Flora-On also utilises a morphological dataset not currently published elsewhere, as well as a number of other quantitative data fields that numerically describe the flowering period, bioclimatic and altitudinal distribution profiles of each taxon (Figure 2).

Morphological data is a compilation of information from different bibliographic sources (Castroviejo 1986–2015, Franco 1971–1984, Franco and Rocha-Afonso 1994–2003) and from direct observation in the field, which includes ca. 15 categorical reproductive and vegetative plant traits, such as colour and number of petals, type of fruit and type of growth. The primary purpose of this trait data was to aid the general public on the identification of taxa, but it is part of the roadmap to enrich the dataset with more traits and make it freely available.

Species altitude profiles and bioclimatic profiles are estimated by applying a kernel density on elevation and bioclimatic data, i.e. the set of elevation and bioclimatic variable values at which each taxon was observed. Elevation data is extracted by crossing taxa occurrence data with the ASTER Global Digital Elevation Model (METI, NASA). Bioclimatic data are extracted from the climatic variables and bioclimatic indices compiled and developed by Monteiro-Henriques et al. (2015).

The Flora-On dataset represents a major contribution to the knowledge of the present distribution of Portuguese and Iberian flora. Despite the lack of information in several parts of the territory, Flora-On dataset constitutes the most complete and up to date source of research-grade occurrence data on the Portuguese flora, since a great concern is put on ensuring the correctness of the data. Other existing nation-wide platforms covering occurrence data of the Portuguese flora have either a partial coverage or do not specifically target validated data. Furthermore, previous data on the Portuguese flora was limited to herbarium and bibliographic sources, which are largely not digitally accessible or accessible only in a very coarse format.

Finally, the Flora-On project has been stimulating the collection of new data on the distribution of species, which has resulted in great improvements in the knowledge of many species. Indeed, the voluntary field work conducted by the collaborators has significantly improved the knowledge about the current status of many rare, protected by national and international legislation, or hardly known species, and several new species not known to occur in Portugal have been recently found.

### Dataset description

**Object name**: Darwin Core Archive Flora-On: occurrence data of the flora of mainland Portugal

**Character encoding**: UTF-8

**Format name**: Darwin Core Archive format

**Format version**: 1.0

**Distribution**: http://flora-on.pt:8080/ipt/archive.do?r=flora-on

**Publication date of data**: 2016-02-19

**Language**: Portuguese

**Licences of use**: This work is licensed under a Creative Commons Attribution Non Commercial (CC-BY-NC) 4.0 License.

**Metadata language**: English

**Date of metadata creation**: 2014-12-04

**Hierarchy level**: Dataset

**Used Darwin Core Terms**: id, modified, language, rights, institutionID, collectionID, institutionCode, collectionCode, datasetName, ownerInstitutionCode, basisOfRecord, dataGeneralizations, occurrenceID, catalogNumber, occurrenceRemarks, recordedBy, reproductiveCondition, occurrenceStatus, eventDate, year, month, day, country, countryCode, county, municipality, decimalLatitude, decimalLongitude, geodeticDatum, footprintWKT, footprintSRS, identifiedBy, dateIdentified, scientificName, higherClassification, kingdom, phylum, class, order, family, genus, specificEpithet, infraspecificEpithet, taxonRank, scientificNameAuthorship, nomenclaturalCode.

## References

[B1] ASTER (2016) Global Digital Elevation Model (METI, NASA). https://asterweb.jpl.nasa.gov/gdem.asp

[B2] Castroviejo S (Ed.) (1986–2015) Flora Iberica. Plantas Vasculares de la Península Ibérica, e Islas Baleares. Real Jardín Botánico CSIC, Madrid.

[B3] Franco JA (1971–1984) Nova Flora de Portugal (Continente e Açores). Vols. I, II Edition of the Author, Lisbon.

[B4] Franco JA Rocha-Afonso ML (1994–2003) Nova Flora de Portugal (Continente e Açores), Vol. III (I/II/III) Escolar Editora, Lisbon.

[B5] Monteiro-Henriques T Martins MJ Cerdeira JO Silva P Arsénio P Silva Á Bellu A Costa JC (2016) Bioclimatological mapping tackling uncertainty propagation: application to mainland Portugal. International Journal of Climatology 36: 400–411. doi: 10.1002/joc.4357

[B6] Pereira D Pereira P Santos L Silva J (2014) Unidades Geomorfológicas de Portugal Continental. Revista Brasileira de Geomorfologia 15(4): 567–584. http://repositorium.sdum.uminho.pt/bitstream/1822/33835/1/Pereira_RBG_2014.pdf

[B7] Sequeira M Espírito-Santo D Aguiar C Capelo J Honrado J (Coord.) (2010) Checklist da Flora de Portugal (Continental, Açores e Madeira). Associação Lusitana de Fitossociologia (ALFA).

[B8] Tennekes M (2016) TreeMap: Treemap Visualization. R package version 2.4–1. https://CRAN.R-project.org/package=treemap

